# Observations on the Bad Effects Produced by the Use of Mercury in Several Scrofulous Cases

**Published:** 1809-12

**Authors:** Monsieur Fauberge

**Affiliations:** Doctor-Surgeon, Member of the Legion of Honour, &c.


*Observations on the bad Effects produced by the Use of Mercury in several Scrofulous Cases;* by MONSIEUR FAUBERGE, Doctor-Surgeon, Member of the Legion of Honour, &c.

*Observation I.* A Captain of Infantry of the line, had in his infancy some scrofulous suppurating tumours about his neck and on the sternum, which were not cured till the age of puberty.

At the beginning of the revolution he took up arms; I have known him in this situation for these eight years, enjoying the most perfect health. In the sixth year of the republic, he applied to me on account of a chancre which he had been trying to cure by various caustic applications, during the use of which the disease only grew worse.—Van Swieten's solution, fomentations, and mercurial frictions were administered alternately. At the expiration of twenty days of this treatment, the chancre was perfectly cured; but the patient complained soon after of swellings in the neck and in the axilla, which, though not painful, were troublesome. I found these parts full of small fluctuating tumours, but insensible; on touching one with the lancet, a white milky pus issued out. Some days after, others were formed in the hollow of the clavicles, and in the parotids. The breast soon became the seat of a considerable number of these deposits, which were formed slowly, and without the slightest pain: whether they opened spontaneously, or were opened by the lancet, they formed so many suppurating wounds which could not be cicatrised.—I called a consultation of the most respectable medical men of Toulon; M. Crepin, physician; Giraut-Saint Rome, surgeon to the Military Hospital School of that city, were of the number.—On the presumption that this this state might arise from the complication of the venereal virus with the first disease, in the same manner as it might depend on the effect of the mercury on the latter, it was determined to put the patient on a course of the rob d'affecteur. He took six bottles of it, and was no better; we then directed our attention to the scrofula, and were equally unsuccessful. This worthy officer, dragged on a miserable existence for three years, having a great part of his body covered with plasters; he died in a marasmus, at the age of 33

*Observation II.*—Being at Malta, in the seventh year of the republic, I was consulted by a Captain, who, for a gonorrhœa, accompanied by a slight swelling in the inguinal glands, had been put under a course of mercurial frictions of a drachm each. At the fifth friction, the swelling in the groins was much increased, and the parotids became considerably larger. It was at this period I first saw the patient; he informed me he had been subject to similar swellings in his infancy; but with age and the exercise of hunting, had completely got the better of them; that he believed his constitution had the complaint from his parents. Presuming, from this account, the existence of scrofula, I treated my patient accordingly; the success confirmed my opinion.

*Observation III.*—A quarter-master of the sixth brigade of Infantry, entered the Venereal Hospital, at Malta, in the winter of the eighth year of the republic, to be treated for a chancre and bubo. At the eighth friction, twenty days after his admission into the hospital, he was in a most pitiable state; the parotids and the whole neck were covered with scrofulous tumours; the old cicatrices which had been formed in his infancy in those parts opened, and a considerable tumour was formed in the joint of the right foot. In this situation he was brought into the hall of the great hospital, of which I was physician-general; by degrees the tumours were brought to a point. That on the foot discovered a deep and considerable caries in the joint, which no applications could check.

The emaciated state of the patient, and the absolute want of good provisions, which we then experienced, on account of the blockade by the English, prevented my attempting the amputation; in other respects, I had little hopes of success from this operation.—But the severe pains constantly suffered, the œdema which reached the upper part of the leg, and the entreaties of the patient to relieve him of so painful a limb, as well as a constant watchfulness, at last prevailed. After the amputation, I made a large issue in the arm; the patient soon regained his rest and appetite. The very interesting situation in which I found this person, induced me to provide him with little comforts from my own table; at the end of two months he was completely cured. I met him four years since at Nogent Sur-Seine, full of health and gratitude.

*Observation IV.*—An officer, 36 years of age, who had been subject in his infancy to scrofulous tumours in his neck and knees, and which he had not felt for twenty years, had, during a campaign in Prussia, contracted a venereal affection. Honour and a love of his duty, retained him with his company, notwithstanding the inconvenience he experienced from forced marches in a country without resources; but the fatigue he endured at Pultuks, obliged him to repair to Varsovia for relief. The mercurial treatment he at first underwent, removed the venereal symptoms; but the parotids increased; some old cicatrices opened a-fresh, and produced callous and foul ulcers. Not being able to continue the campaign, he returned to France, where I cured him by the sole use of chalybeates and one drachm of the muriate of soda, night and morning, dissolved in three ounces of pure water.

